# Structure modulated charge transfer in carbon atomic wires

**DOI:** 10.1038/s41598-018-38367-9

**Published:** 2019-02-07

**Authors:** A. Milani, V. Barbieri, A. Facibeni, V. Russo, A. Li Bassi, A. Lucotti, M. Tommasini, M. D. Tzirakis, F. Diederich, C. S. Casari

**Affiliations:** 1 Micro and Nanostructured Materials Laboratory - NanoLab, Department of Energy, Politecnico di Milano via Ponzio 34/3, I-20133 Milano, Italy; 2 Department of Chemistry, Materials and Chem. Eng. ‘G. Natta’, Politecnico di Milano Piazza Leonardo da Vinci 32, I-20133 Milano, Italy; 3 Laboratory of Organic Chemistry, Department of Chemistry and Applied Biosciences, ETH Zurich, Vladimir-Prelog-Weg 3, 8093 Zurich, Switzerland

## Abstract

sp-Hybridized carbon atomic wires are appealing systems with large property tunability. In particular, their electronic properties are intimately related to length, structure, and type of functional end-groups as well as to other effects such as the intermolecular charge transfer with metal nanoparticles. Here, by a combined Raman, Surface Enhanced Raman Scattering (SERS) investigation and first principles calculations of different N,N-dimethylanilino-terminated polyynes, we suggest that, upon charge transfer interaction with silver nanoparticles, the function of sp-carbon atomic wire can change from electron donor to electron acceptor by increasing the wire length. In addition, the insertion into the wire of a strong electrophilic group (1,1,4,4-tetracyanobuta-1,3-diene-2,3-diyl) changes the electron-accepting molecular regions involved in this intermolecular charge transfer. Our results indicate that carbon atomic wires could display a tunable charge transfer between the sp-wire and the metal, and hold promise as active materials in organic optoelectronics and photovoltaics.

## Introduction

Carbon atoms with sp-hybridization arrange in linear structures, constituting the ideal infinite 1D carbon wire (i.e. the carbyne)^1,2^. Carbyne attracts interest as a new carbon allotrope^3–6^ and for its predicted fascinating properties, such as exceptional mechanical stiffness, electron mobility, thermal conduction, as well as peculiar strain induced metal-to-insulator transition, ballistic transport, and nonlinear optical properties, as recently pointed out by a number of works^7–16^. Most of these properties are a direct consequence of the π-electron conjugation resulting from the linear configuration and the hybridization state. In finite carbon-atom wires (CAWs), the properties can be modulated by adjusting the length of the sp-carbon chain and the type of the end-group^17^. Such opportunity fits with experimentally available systems whose length ranges typically from two carbon atoms to a few tens^18^, with the record length of 44 atoms reached by R. Tykwinski^19^. As a recent example of the potential exploitation of such tunability, optical supermultiplexing and barcoding have been successfully demonstrated with a set of finite polyynes with engineered length and termination^20^. In addition to the property modulation, the end-group can be used to control the structural and electronic configuration inducing either the polyyne-like structure^21^ (i.e. alternating triple and single bonds in a dimerized geometry) or the cumulene-like structure^22–25^ (i.e. sequence of double bonds in equalized geometry).

Recently, rational chemical synthesis successfully provided many stable systems with controlled size and type of termination^17–19,22–24,26^. The proper choice of terminating groups showed to be a promising strategy to improve stability against cross-linking reactions. Typical terminations include sp^2^ aromatic groups able to ensure steric hindrance and long-term stability in ambient conditions opening new possibilities for understanding structure-property relationship through a detailed investigation of these systems.

Vibrational spectroscopy has shown all its potential for detecting and investigating structural and electronic properties of sp-carbon systems^27^. Raman spectroscopy can provide unambiguous insights on the structure and properties of sp-carbon molecules and thin films due to its extreme sensitivity to carbon hybridization, π-conjugation, and local order^1,27–29^. In addition, Surface Enhanced Raman Scattering (SERS) gives access to the study of the interaction with metal nanoparticles and charge transfer processes^17,30–32^.

SERS exploits the local enhancement of the electric field upon excitation of surface plasmons by incident photons (i.e. electromagnetic SERS effect) or due to a charge transfer and the formation of a complex between the investigated molecule and the metal nanoparticle (i.e. chemical SERS effect). In the case of H-terminated polyynes, we showed an enhancement factor of 10^6^ with respect to normal Raman^30,31^. The chemical SERS mechanism has been used to unveil a charge transfer followed by a structural reorganization of the sp-wire towards a more equalized configuration, as shown in the case of phenyl-terminated polyynes^17,32^. Understanding in detail the direction of charge transfer and the distribution of the excess charge is a fundamental step to deepen the knowledge of these systems and to design new systems for possible future applications. In this framework, the role of conjugation in determining the vibrational properties and the occurrence of charge transfer still needs to be further clarified. In fact, so far, charge transfer in polyyne-like systems has been outlined for poly-dispersed sp-carbon chains only^17,30–32^ and the physicochemical insight into the role of chain length and degree of π-conjugation is still missing. In addition, the effect on the charge distribution when a molecular group is inserted between the chain and its termination has been never investigated, up to our knowledge.

**Figure 1:**
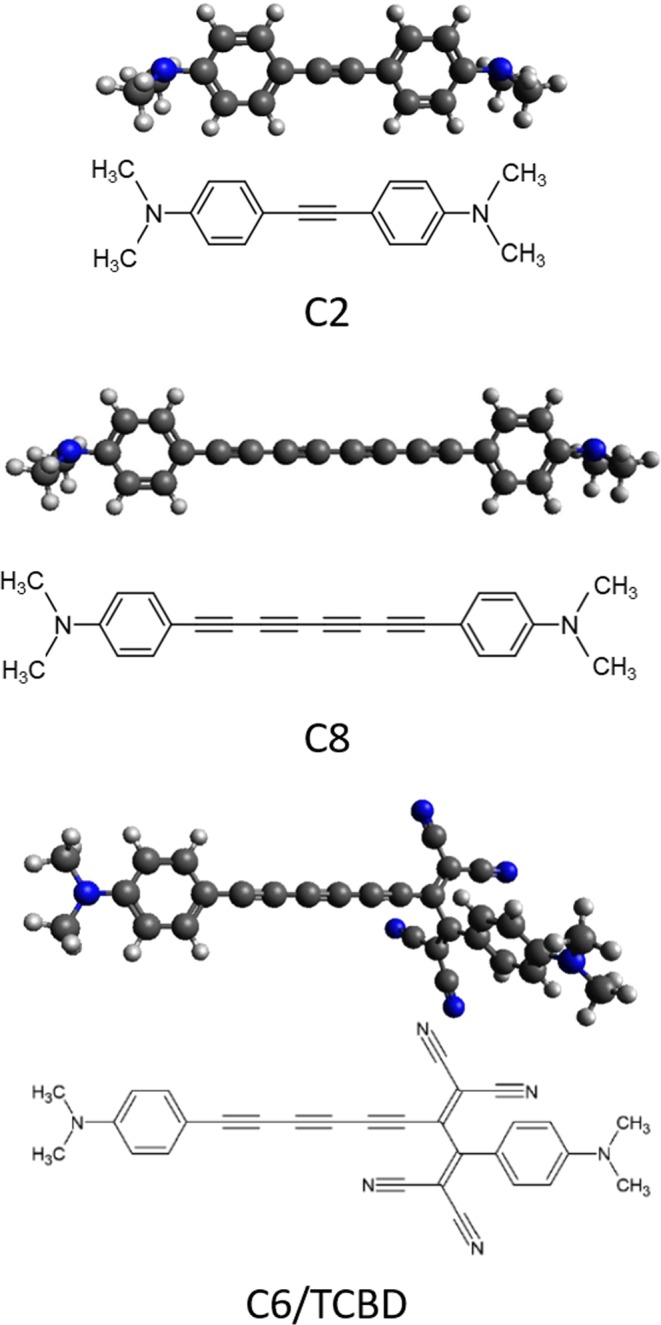
Chemical structures and DFT-calculated molecular conformations of the investigated systems: C2, C8, and C6/TCBD.

Here, we investigate N,N-dimethylanilino-terminated polyynes with selected structure (in terms of sp-chain length and termination, see Fig. 1) as model systems to shed light on the role of π-electrons conjugation and the occurrence of intermolecular charge transfer between CAWs and metals. The selected wires have the same termination and different sp-chain length (2, 6, and 8 carbon atoms). In one case, a tetracyanobuta-1,3-diene-2,3-diyl (TCBD) group was inserted into the sp-wire by [2 + 2] cycloaddition–retroelectrocyclization (CA–RE) reaction^26,33^ before reaching the termination.

We show by Raman, SERS, and density functional theory (DFT) calculations that the wire length and conformation affect conjugation and hence the charge transfer from/to the metal. The interaction of the investigated CAWs with metal nanoparticles can be interpreted as an intermolecular charge transfer whose direction depends on the wire length, moving from an electron acceptor to an electron donor system. Interestingly, the insertion of the TCBD group seems to lead to a different distribution of the transferred charge changing from parallel to perpendicular with respect to the sp-wire direction.

## Results

The three investigated systems consist of polyyne-like carbon-atom wires (see Fig. 1) with one to four acetylenic units (i.e. 2 to 8 sp-carbon atoms) terminated by N,N-dimethylanilino (DMA) groups (called hereafter C2 and C8, respectively) and a system with a single 1,1,4,4-tetracyanobuta-1,3-diene-2,3-diyl (TCBD) group perpendicular to the sp-wire, composed by three acetylenic units (i.e. 6 sp-carbon atoms) and DMA end groups (called hereafter C6/TCBD)^26,34^.

**Figure 2:**
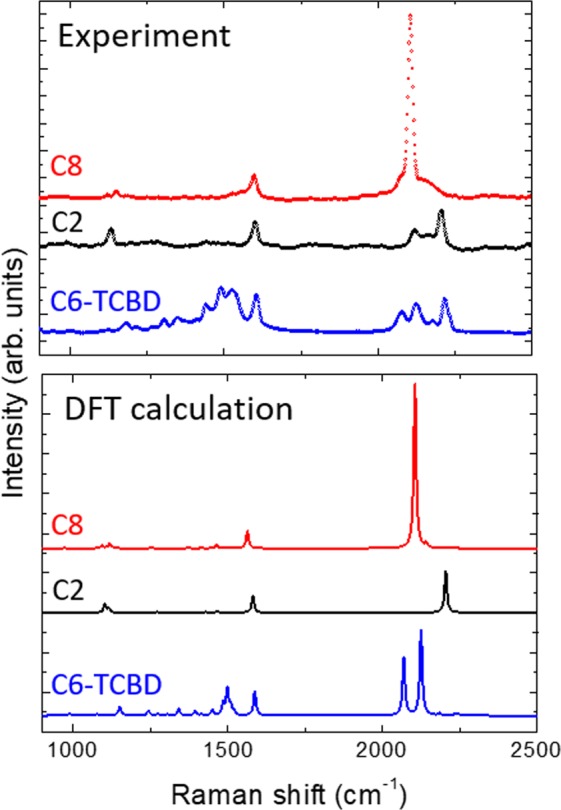
(Top) Experimental micro-Raman spectra of films of C2, C8, and C6/TCBD on glass taken at 514.5 nm excitation wavelength. (Bottom) DFT-calculated Raman spectra of C2, C8, and C6/TCBD.

The investigated systems are stable when deposited as films on different substrates as demonstrated by Raman spectra in solution and in films (see Fig. 2, Figs S1 and S2). Raman spectra of the CAWs deposited on glass are in nice agreement with DFT calculations of the Raman response (Fig. 2). Three characteristic spectral regions are present: a peak at about 1600 cm^−1^, common to all samples, is assigned to the carbon-carbon stretching of the phenyl groups; the peaks below 1200 cm^−1^ are assigned to ring-breathing modes, CH bending vibrations or other CCC bending modes. The Raman features observed in the 2000–2300 cm^−1^ spectral region are marker bands of the sp-carbon chains. Raman spectra of C2 and C8 show almost single peaks in all the mentioned regions, whereas the more complex molecular structure of C6/TCBD give rise to a much higher number of peaks with respect to the other DMA-terminated systems.

Focusing on the spectral region relevant for sp-carbon we observe that the peak wavenumber downshifts by more than 100 cm^−1^ (from 2206 cm^−1^ to 2117 cm^−1^) as the number of sp-hybridized carbon atoms increases (i.e. from C2 to C8, see Fig. S3). For C8, the most intense peak corresponds to the so called ECC (effective conjugation coordinate) mode of conjugated systems, i.e. the collective stretching of triple bonds and shrinking of single bonds in the chain^35^. C8 has an additional weak peak at higher wavenumber (≈2150 cm^−1^). This is predicted by DFT, and it corresponds to the longitudinal oscillation of the bond length alternation with a relative phase with respect to the ECC (α-mode); it is assigned to the so-called β-mode of vibration^35,36^. Finally, for the most conjugated molecule (C8) we have observed overtones of the sp-carbon related peaks at about 4200 and 4135 cm^−1^ in very intense Raman spectra acquired in correspondence of a coarse aggregate (see Fig. S3).

For C6/TCBD, the analysis of the band in the sp-region, compared with the DFT-simulated spectrum, shows comparable patterns. The peaks at 2075, 2126, and 2177 cm^−1^ in the experimental spectrum can be assigned to different combinations of longitudinal CC stretching modes still localized on the sp-hybridized chain^37^. In particular, the peak at 2126 cm^−1^ is assigned to the ECC-mode (see Fig. S4). Instead, the last peak at 2216 cm^−1^ and the shoulder at 2231 cm^−1^ respectively originate from C≡N bonds stretching in the TCBD substituent. By comparing experiments with DFT calculations, a very marked difference is visible in the relative intensity of these peaks. Taking as reference the intensity of the signal corresponding to stable terminal C≡N bonds, we observe that the intensities of the sp-chain modes in the experimental spectrum are significantly lower than the DFT ones. This can be due to the well-known tendency of current DFT functionals in overestimating π-electron conjugation, including an overestimation of the derivatives of the polarizability tensor and thus of the Raman activity of the normal modes localized on the conjugated molecular domains^24,34^. No substantial differences are seen for Raman spectra taken at different laser wavelength (i.e. from 1064 nm (FT-Raman) to 457 nm, see Fig. S5).

**Figure 3:**
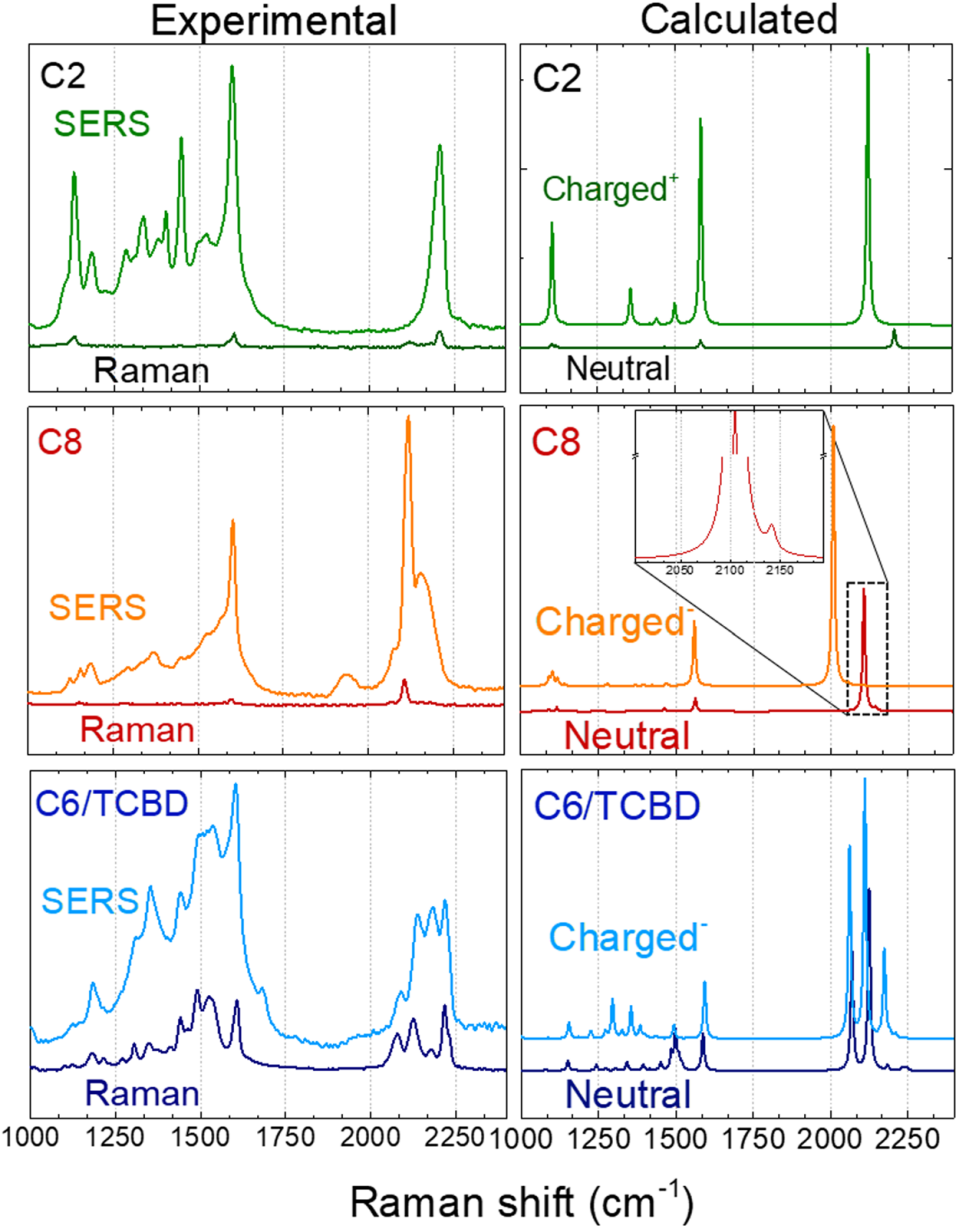
(Left) Experimental solid-state SERS spectra at 514.5 nm of C2, C8, and C6/TCBD deposited on SERS-active substrate (Ag nanoislands on glass). Micro-Raman (514.5 nm) spectra are reported for comparison. (Right) comparison of DFT computed Raman spectra of neutral, positively charged C2 and negatively C8, and C6/TCBD (see text for discussion).

To investigate a possible interaction of these systems with metal nanoparticles and to unveil the occurrence of charge transfer effects, solid-state SERS measurements were performed on polyynic film deposited on SERS-active substrates made of silver nanoislands. For all the investigated systems, the SERS spectra (Fig. 3) show prominent molecular features and new intense peaks arising in the region between 1150 and 1550 cm^−1^. These bands originate from vibrational modes involving C-N stretching vibrations, as well as N-C-H and C-C-H bending vibrations mainly localized on the end groups.

In SERS, features in this region seem to protrude from a broad underlying band. This pattern can be due to different possible effects. On one side a broadening of the peaks is typical of SERS spectra and we expect the presence of spurious sp^2^-carbon signal due to carbon contaminations enhanced by SERS. On the other hand, the generation of a band due to crosslinking and partial sp - to - sp^2^ conversion of carbon wires cannot be excluded a priori, even though not expected due to the high stability of the investigated systems^28–31^.

Focusing on the sp-carbon region (1800–2200 cm^−1^), a single very intense and broad line can be identified in the SERS spectrum for the C≡C bond stretching in C2 and for the ECC mode in C8 (located at 2207 cm^−1^ and 2117 cm^−1^, respectively). At difference with C2, for C8 the region of the spectrum associated to sp-carbon shows an important peculiarity: a completely new band appears at about 1935 cm^−1^, well below 2000 cm^−1^, i.e. at about the typical vibrational frequency for cumulene-like wires with almost equalized bond lengths. SERS spectrum of C8 also shows a shoulder of the main peak which may be related to the enhancement of a high frequency feature that is actually predicted by DFT calculation and assigned to the β mode (see inset of Fig. 3). Such feature is not observed in the normal Raman spectrum probably due to its low intensity.

For C6/TCBD we observe a lower SERS enhancement with respect to other systems with no major change in number or shape of features below 1600 cm^−1^, except for peak broadening. Nevertheless, some change in the relative intensities of peaks composing the band between 2050 and 2250 cm^−1^ can be observed. In particular, the peaks located at 2126, and 2177 cm^−1^, which are related to vibrational modes inside the sp-carbon chain, show a preferential enhancement.

## Discussion

To identify the origin of the new bands and the other variations observed in the sp-carbon region of conjugated systems, we compare the SERS with the normal Raman spectra. The additional band between 1900 and 2000 cm^−1^ present in the spectrum of molecule C8 can be associated to a charge transfer between sp-carbon wires and metal nanostructures, which agrees with previous works^17,32^. It has been already demonstrated, indeed, that charge transfer can induce a change in the electronic structure, decreasing the bond length alternation. Smaller bond length alternation points to more equalized structure towards cumulene-like systems which have lower-frequency Raman features for the same wire length. Previous works show that charge transfer is preferentially driven from the metal nanoparticle to the carbon chain, which thus gain a partial negative charge^17,32^. For the C8 molecule, the SERS band is weaker than the Raman peak, possibly because of the small number of molecules being able to directly interact with the active substrate. DFT calculations support this interpretation showing a good agreement with calculated Raman spectrum of negatively charged (−e) structures, according to the behavior displayed by CAWs capped with phenyl or bi-phenyl groups^17,32^.

The case of C2 seems not to follow this charging scheme and no new bands appear in C2 in the frequency range associated to stretching modes of the sp carbon chain. We attribute this to the very limited conjugation of this molecule. In fact, the two sp-hybridized carbon atoms in the chain of C2 are not enough to set up an effective π-conjugation path since no overlap between adjacent π-bonds can take place as in longer chains. It should be noticed that the SERS spectrum of C2 reveals significant changes in the frequency range below 1600 cm^−1^, where several intense bands arise and give a spectral pattern significantly different with respect to the Raman spectrum. As noticed before, vibrational modes active in this region mainly involve the terminal group, indicating that charge transfer is affecting preferentially the end-groups, which act as electron donors. Indeed, based on DFT calculations of the positively charged C2, the new bands appearing below 1600 cm^−1^ are assigned to CC stretching coupled to CH wagging vibrations of the phenyl groups (1356 cm^−1^), and to CN stretching coupled to CH wagging vibrations (1498 cm^−1^). These modes are very weak for the neutral C2. This is different from the case of the intense band at 1108 cm^−1^, which is assigned to the ring breathing mode of the phenyl groups both in charged and neutral C2.

DFT calculation of the Raman spectrum of the negatively charged C2 molecule has a pattern that cannot describe the SERS spectrum, ruling out the occurrence of charge transfer from the metal to the CAW. In fact, for negatively charged C2 the calculated spectrum has no peak at 1600 cm^−1^ and does not predict the intensification of the low frequency region (See SI, Fig. S6). On the other hand, DFT calculations show good agreement with the positively charged C2 system and confirm the occurrence of new intense bands below 1600 cm^−1^ in the SERS spectrum of C2 and the correct peak position of the intense band at 1600 cm^−1^ due to phenyl groups (Fig. S6). Calculations predict a slight shift of the main sp-chain related peak (C≡C stretching mode) towards lower frequency, which is not clearly seen in experiments as a new peak. To explain this difference, we underline that simulations consider one entire electron charge transfer, whereas a partial charge transfer may be involved. This can be more relevant for short CAWs, in which is more difficult to distribute the excess charge. In addition, the experimental SERS spectrum of C2 shows a broader sp-chain related peak that is compatible with a convolution of features originating on one side from charged C2 species interacting with metal nanoparticles and, on the other, from neutral C2 not close enough to the metal to undergo charge transfer.

This evidence seems to suggest that C2 displays an opposite behavior with respect to C8 in the charge transfer process, preferring to act as an electron donor. To support this interpretation, we have calculated the formation energy (E_ion_) of the metal/CAW system in the two charge-transfer configurations, namely Ag^+^/CAW^−^ and Ag^−^/CAW^+^ (see Methods section for details).

**Figure 4:**
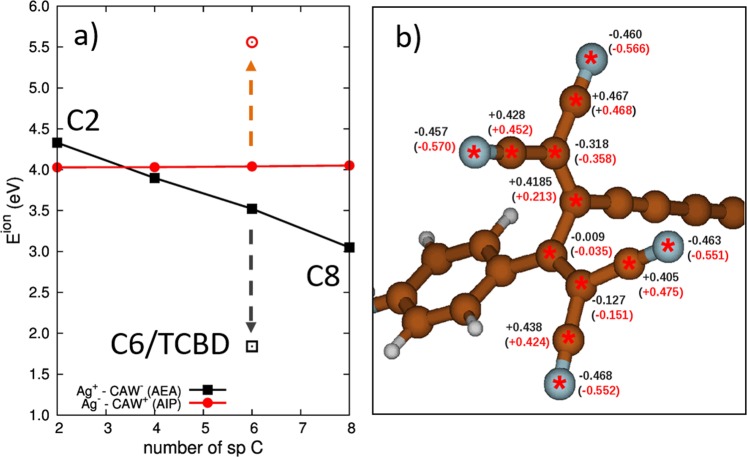
(**a**) Plot of the DFT computed values of E_ion_ (see Methods section) for the systems investigated (**b**). Balls and sticks model of the DFT-simulated charge distribution (CHELPG partial atomic charges) of the TCBD moiety of C6/TCBD. Numbers written in black are the partial atomic charges of the neutral molecule, while the red ones are those of the negatively charged molecule.

The numerical values of the computed energies are plotted in Fig. 4a (and reported in Table S1). There, to further verify the observed trend, we also consider the intermediate case of the C4 and C6 molecules (not experimentally available here). These data confirm the interpretation suggested above: E_ion_ inverts upon moving from C2 to C8, and the intermolecular charge transfer for C2 is energetically favoured when occurring from the CAW to the metal substrate. This explains the fact that the Raman spectrum of the positively charged C2 molecule is the one which better accounts for the experimental SERS spectrum.

The shortening of the sp-chain length increases the energy for negative charging making the system more prone to donate the charge to the metal nanoparticle. This also agrees with chemical intuition, which suggests that sp-carbon atoms play the role of electron acceptors: indeed, just increasing the length of the chain from 2 to 4 sp-carbon atoms is already enough to turn the CAW from an electron donor to an electron acceptor, and this tendency consistently increases in longer molecules with a higher number of sp carbon atoms.

Considering now the case of C6/TCBD, based on the previous discussion we would expect that the SERS spectrum of this molecule should behave similarly to C8, with the appearance of a new contribution at 2000 cm^−1^ (or just below) as a result of negative charge transfer. However, both the SERS spectrum and the DFT calculations of the negative species do not show appreciable changes in the frequency region above 2000 cm^−1^, while they both describe some pattern variation below 1600 cm^−1^. The fact that DFT calculations of negatively charged C6/TCBD do not indicate much changes in the wavenumber of the longitudinal CC stretching modes of the sp chain reveal more intriguing details. C6/TCBD is chemically more complex than C6, due to the presence of the TCBD group, which “crosses” the sp-wire. There, the cyano functional groups (C≡N) are prone to form dative bonds with metal surfaces and, therefore, they may compete with the π-orbitals of the sp-chain when C6/TCBD interacts with metal nanostructures. The calculations of E_ion_ (see Table S1 and Fig. 4a) indicate that C6/TCBD is very likely to act as an electron acceptor, even more than the C6 molecule with the same number of sp-carbon atoms. This suggests that the C≡N bonds of TCBD further promote the occurrence of negative charge transfer from the metal substrate to the CAW. The stronger electron acceptor behavior of C6/TCBD seems to contradict the lack of a significant modulation of the CC stretching modes along the sp-chain. To solve this issue, we analyzed the atomic charge distribution by computing partial atomic charges for the neutral and negatively charged C6/TCBD molecule (see Methods Section). The values of the atomic charges are reported in Fig. 4b, focusing on the TCBD group containing the C≡N bonds. By computing the total charge on the atoms marked with a red “*” in the neutral C6/TCBD and its anion, a value of −0.0555 e is found for the neutral case, whereas a value of −0.751 e is obtained for the negatively charged case. This indicates that about −0.70 e of the excess unitary electron charge of the anion is localized at the TCBD moiety, whereas only the remaining −0.3 e is distributed in the rest of C6/TCBD (including the sp carbon domain). In other words, the C≡N groups of C6/TCBD, and the sp^2^ carbon network connecting them, act as preferential acceptors of the negative charge, which reduces the modulation of both the structure and the Raman activity of the sp carbon chain. This is different from the case of phenyl capped CAW^17^ or the C8 molecule, where the negative charges preferentially localize on the sp carbon chain, strongly modulating both the molecular structure and the CC stretching Raman modes of the sp carbon chain.

Based on these findings, in C6/TCBD we can identify two distinct and independent charge transfer paths. The first path is along the sp-hybridized carbon chain, as for C8, while the other is represented by the TCBD substituent. The two are separated by an angle of about 121.4°^38^, along which charge transfer is impeded. For this reason, upon interaction with metal nanoparticles, the charge will tend to be delocalized along the TCBD group, thus preventing the electron flow of the transferred charge along the sp-carbon domain.

**Figure 5:**
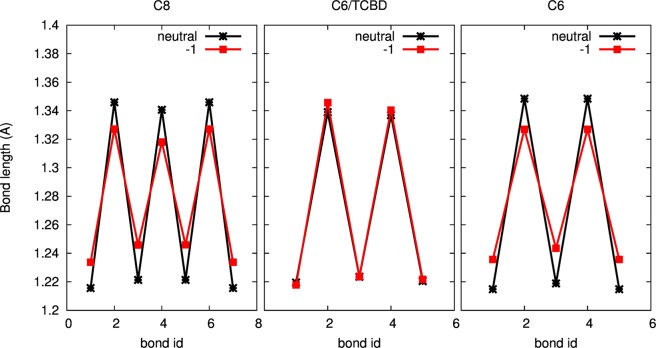
DFT-computed lengths of sp-carbon bonds in C8, C6/TCBD, and C6 for neutral and negatively charged species.

The interpretation discussed above of the SERS spectrum of C6/TCBD through the computed Raman spectrum of the negatively charged model is also supported by the analysis of the bond length alternation (BLA – Table S1). As reported in previous investigations on CAWs terminated by sp^2^-conjugated groups^17,32^, negatively charged molecules are characterized by lower BLA, which indicates that the charge transferred from the metal to the CAW mainly affects the sp-carbon domains, and promotes the equalization of bond lengths and the red-shift of the ECC band. This is explained by the connection existing between the position of the ECC Raman mode and BLA^39^. This kind of behavior is observed here also for C8: the new band observed in SERS at about 1930 cm^−1^ is red-shifted by 180 cm^−1^ with respect to its Raman counterpart. This is consistent with DFT calculations of negatively charged C8, whose BLA is lower than that of neutral C8 (0.0842 Å vs. 0.1257 Å). As for C6/TCBD, the lack of wavenumber shift of SERS vs. Raman features in the sp-CC stretching region, which is consistent with the similar position of the peaks computed by DFT in the neutral and negatively charged model, suggests that the charge transferred from the metal does not affect the sp-carbon domains. In this scenario BLA would not change in the negatively charged model with respect to the neutral one, which is exactly the case (BLA = 0.1167 Å in neutral C6/TCBD; BLA = 0.1220 Å in negatively charged C6/TCBD). This is evidenced by the trends of bond lengths of C8, C6/TCBD and C6 reported in Fig. 5. In fact, the comparison between C6/TCBD and C6 clearly shows the effect of the TCBD group in moving the excess charge away from the sp-chain and thus strongly affecting the BLA. Therefore, the complete picture given by the analysis of Raman spectra, atomic charges and BLA of C6/TCBD model confirms the different charge transfer effect which is here observed, where TCBD group and not the sp-carbon domains acts preferentially as an electron acceptor in the intermolecular charge transfer with silver.

## Conclusions

N,N-dimethylanilino-terminated polyynes have been used as model systems to investigate intermolecular charge transfer phenomena on silver surface by means of Raman/SERS experiments and DFT calculations. We have addressed the dependence of charge transfer upon sp-carbon chain length and functionalization of the sp-chain by a TCBD group.

Based on our results we suggest that the length of the sp carbon chain can have a significant role in determining the direction of the charge transfer with respect to the interacting metal nanoparticle. Our theoretical results show that in a 2-atoms chain (i.e. one acetylenic unit) the electron acceptor strength is low, due to the limited number of electronegative sp-carbon atoms, and the system is energetically favored to act as an electron donor to silver. Consistently, we propose an interpretation of SERS spectra based on the DFT model of a positively charged wire. By increasing the number of sp-carbon atoms, CAWs become electron acceptors and withdraw electronic charge from the silver substrate. Correspondingly, the SERS spectra of longer chains can be interpreted by DFT models of anionic species. The case of C6-TCBD suggests that the addition of electron accepting groups (i.e. TCBD) within the sp-carbon chain of CAW generates another charge transfer path, which competes with the sp-carbon domain.

Further investigations based on single molecule spectroscopy on other CAWs terminated by donor/acceptor groups may be helpful to deepen the understanding of the charge transfer phenomena in such systems.

These findings not only shed light on physicochemical phenomena in CAWs and related systems, but, they can also impact the investigation of new carbon-based materials. The functionalization of the basic sp-chain structure by chemical design, modulates the response of the sp-carbon domains by means of charge transfer and interactions with metals^17,30–32,40^. This parallels the modulation of electronic and optical properties (band gap, electron transport properties, optical absorption and others) for potential technological applications. Indeed, these molecular properties play a role in the tuning of the insulating/semiconducting/metallic behavior of CAWs and their detailed investigation may open new directions in the science and technology of this class of materials.

## Methods

DMA-terminated polyynes C2^41^ and C8^38^ were prepared according to procedures described previously in literature. The polyyne with TCBD group interrupting the chain was obtained by thermal [2 + 2] cycloaddition-retroelectrocyclization reaction of the corresponding DMA-terminated polyyne with tetracyanoethylene^33^. Details of the synthesis procedure have been reported in ref.^26^.

Sample solutions were obtained by dissolving the DMA-terminated polyynes in dichloromethane (DCM) at 10^−3^ M. After substrate cleaning through a 5 minutes ultrasonic bath in 2-propanol, solid-state samples were produced by casting a single drop (40 mm^3^) of each sample solution on silicon and glass substrates for conventional Raman analysis and on SERS-active substrates for SERS experiments (microscopy images of the films are shown in Fig. S1). In order to avoid solvent entrapment and photodegradation, all samples were dried in vacuum and preserved from direct light exposure.

Solid-state Raman and SERS spectra were acquired with a Renishaw InVia Raman Microscope equipped with an Ar + laser, providing 514.5 nm and 457 nm excitation lines. Being the sample deposits not homogeneously distributed on substrates, local measurements were obtained by focusing the laser beam through a 50x objective (focal spot size of ~1 μm). Laser irradiation power was set at 1 mW and each measure is composed by 3 to 10 (depending on sample stability) 10 s-lasting acquisitions. Protracted laser irradiation caused damages in the deposits, inducing amorphization; this problem was limited by automatic 50% enlargement of the spot size. Luminescence background (very intense for these particular systems) was removed from spectra by subtraction of a straight line or a coarse polynomial fit of the background signal. In-solution FT-Raman analysis was performed with a Nicolet NXR9650 spectrometer equipped with a Nd-YVO4 solid state laser providing a 1064 nm excitation line (spot size: 50 μm); spectra of pure solvents were also recorded and, when possible, subtracted. Each spectrum is composed as a sum of 512 acquisitions at 0.7 W laser power. These settings allowed to limit luminescence (present also in the NIR region), sample heating and solvent evaporation (DCM has Tb = 40 °C). FT-Raman measures have been acquired also on solid-state samples for comparison.

Solid SERS-active substrates were obtained by vacuum deposition (p = 4–6 · 10^−5^ mbar) of silver nanoislands on soda-lime glass and silicon substrates with an Edwards E306A thermal evaporation system. The optimal equivalent thickness (measured with a quartz microbalance) for our purposes was 3 nm, corresponding to an average islands diameter of (13.2 ± 5.7) nm and a surface plasmon resonance wavelength of about 508 nm (morphological SEM images and UV-vis spectra are shown in Figs S7 and S8). As prepared SERS-active substrates without any sample show Raman features in the sp^2^-carbon region (1200–1600 cm^−1^) and at about 2100 cm^−1^ (see Fig. S9). This is due to the high SERS ability to intensify any contaminant or pollutant present on the surface. In particular, the feature at about 2100 cm^−1^ is in the region of interest of sp-carbon and must be removed before performing the SERS analysis of the samples. We succeeded in developing a protocol based on ultrasonic bath to reduce the spurious sp^2^-carbon signal and to remove completely the features in the sp-carbon region (see Fig. S9) while not affecting the Ag nanoparticles. Such cleaning procedure was adopted for all the SERS-active substrates prior to film deposition.

Density functional theory (DFT) calculations have been performed on the isolated DMA-terminated polyynes, employing the hybrid exchange-correlation functional PBE0^42^ together with cc-pVTZ basis set by using the Gaussian09 code^43^. This combination proved indeed to give a very good prediction of structural and spectroscopic properties of many different CAWs^17,24,32,39,44,45^. When comparing theoretical and experimental spectra, a frequency scaling factor has been adopted (0.9488 for C2, 0.9376 for C8, and 0.9499 for C6/TCBD) by considering the experimental value of carbon-carbon stretching mode in the phenyl ring at about 1600 cm^−1^ as a reference band. Based on previous investigations, the interpretation of SERS spectra based on DFT calculations has been carried out by considering charged molecules as models: in order to evaluate in which direction the charge transfer between the metal nanoparticle and the polyyne is energetically favoured, the E_ion_ energy value has been computed as^17,32^:

$${{\rm{E}}}_{{\rm{ion}}}={\rm{IP}}-{\rm{EA}}$$

where IP is the ionization potential of the species acting as electron donor and EA is the electron affinity of the species acting as the electron acceptor in the charge transfer.

E_ion_ is defined as work required for the formation of charged species in the two cases of charge-transfer of one electron from the Ag to the CAW (Ag^+^/CAW^−^) or from the CAW to the Ag (Ag^−^/CAW^+^). The lower values of E_ion_ indicates the most probable process. The values of IP and EA of CAWs have been calculated respectively as the energy difference between the positively charged(+1) and the neutral molecule (AIP–Adiabatic Ionization Potential) and as the energy difference between the negatively charge (−1) and the neutral molecule (AEA – Adiabatic Electron Affinity). The experimental values of 4.6 eV (IP) and −1.30 eV (EA) have been used for the Ag^32^. The evaluation of partial atomic charges of C6/TCBD (see Fig. 4b) has been done according to the CHELPG scheme^46^ as implemented in Gaussian09.

## Supplementary information

Supplementary Information

## Data Availability

The datasets generated during and/or analyzed during the current study are available from the corresponding author on reasonable request.
