# Supplementary material for: Structure modulated charge transfer in carbon atomic wires
Source: Sci Rep. 2019 Feb 7;9:1648. doi: 10.1038/s41598-018-38367-9 (PMC6367501; doi:10.1038/s41598-018-38367-9)
Supplement: Supplementary file 1 — Supplementary Information [file 41598_2018_38367_MOESM1_ESM.pdf]

Supplementary Information

Structure modulated charge transfer in carbon atomic wires

A. Milani[a], V. Barbieri[a], A. Facibeni[a], V. Russo[a], A. Li Bassi[a], A. Lucotti[b], M. Tommasini[b], M. D. Tzirakis[c], F. Diederich[c], C. S. Casari[a,*]

[a] Micro and Nanostructured Materials Laboratory – NanoLab, Department of Energy, Politecnico di Milano via Ponzio 34/3, I-20133 Milano, Italy

[b] Department of Chemistry, Materials and Chem. Eng. 'G. Natta', Politecnico di Milano Piazza Leonardo da Vinci 32, I-20133 Milano, Italy

[c] Laboratory of Organic Chemistry, Department of Chemistry and Applied Biosciences, ETH Zurich, Vladimir-Prelog-Weg 3, 8093 Zurich, Switzerland

*Corresponding author: Carlo S. Casari email: carlo.casari@polimi.it

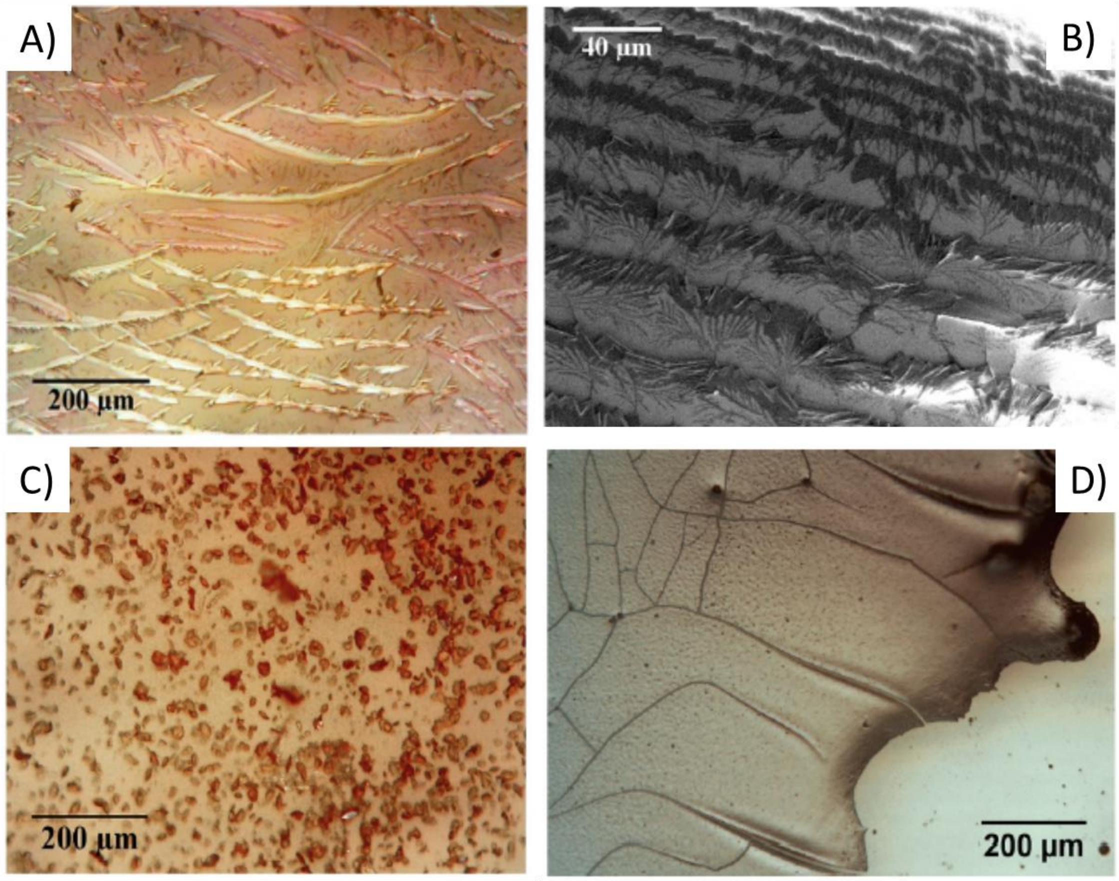

Figure S1: A, B) optical microscopy and SEM image of C2 deposits; C) optical microscopy image of C8; and D) optical microscopy image of C6/TCBD.

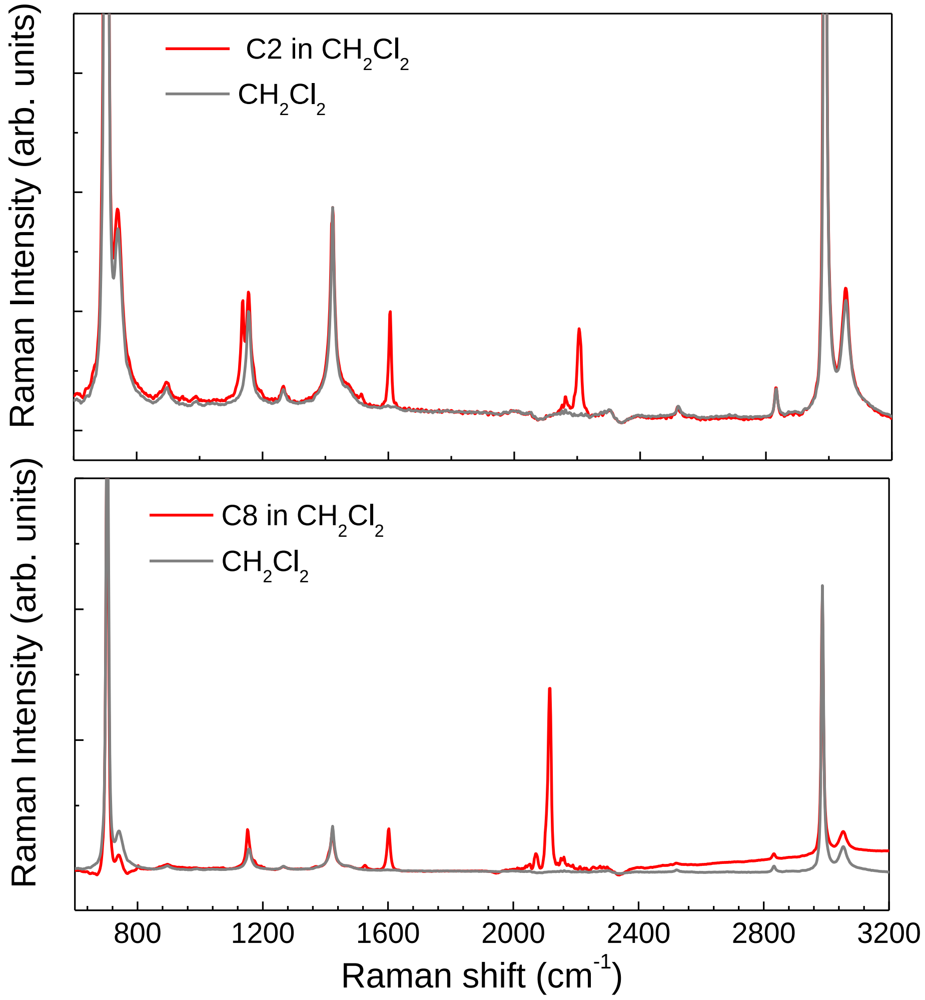

Figure S2: FT-Raman spectra (1064 nm) of C2 and C8 in solution (CH_2Cl_2). The solvent contribution is depicted in grey for comparison.

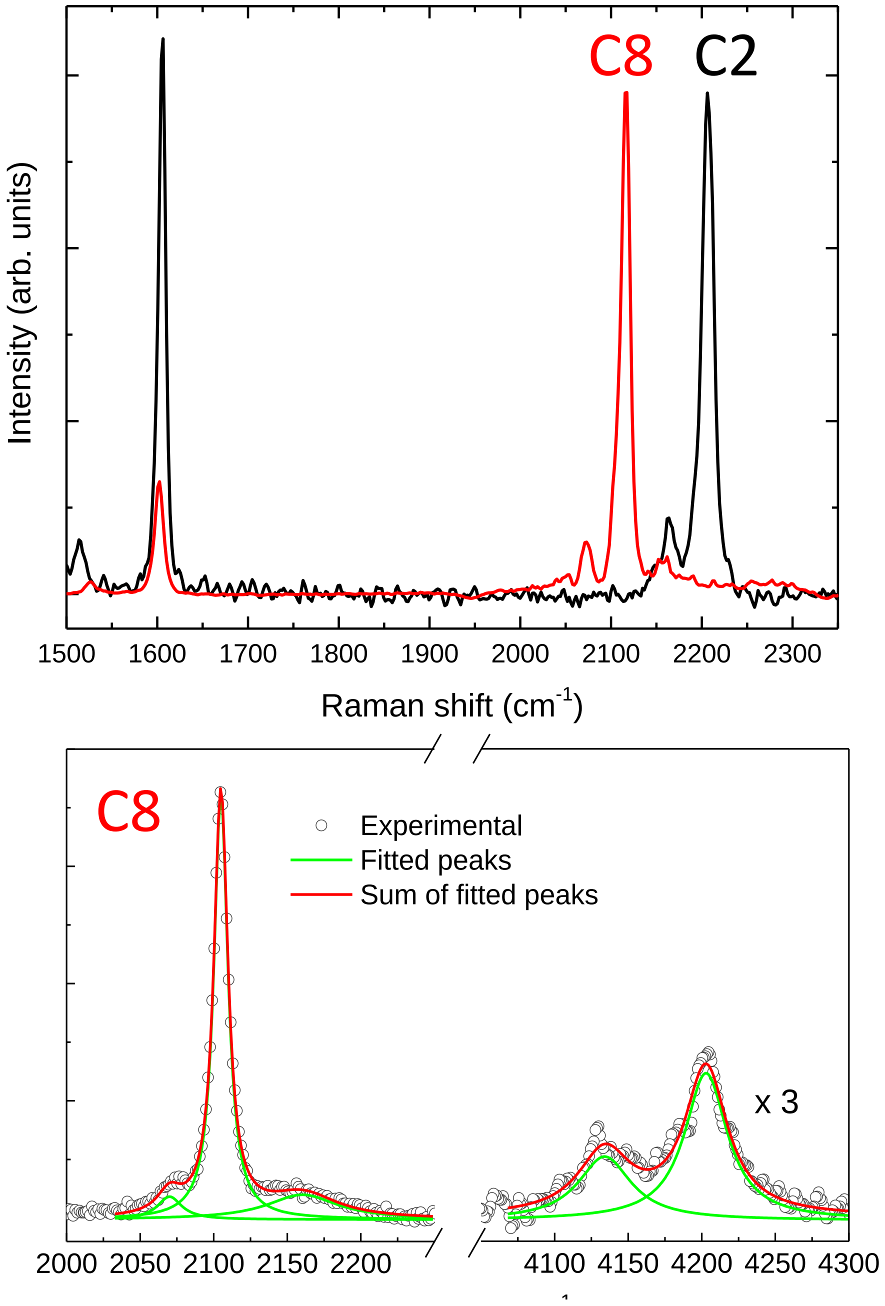

Figure S3: (Top) comparison of FT-Raman spectra of C2 and C8 in solution (CH_2Cl_2; 1064 nm). The contribution of the solvent has been subtracted. (Bottom) fit of ECC mode and second order Raman region for C8.

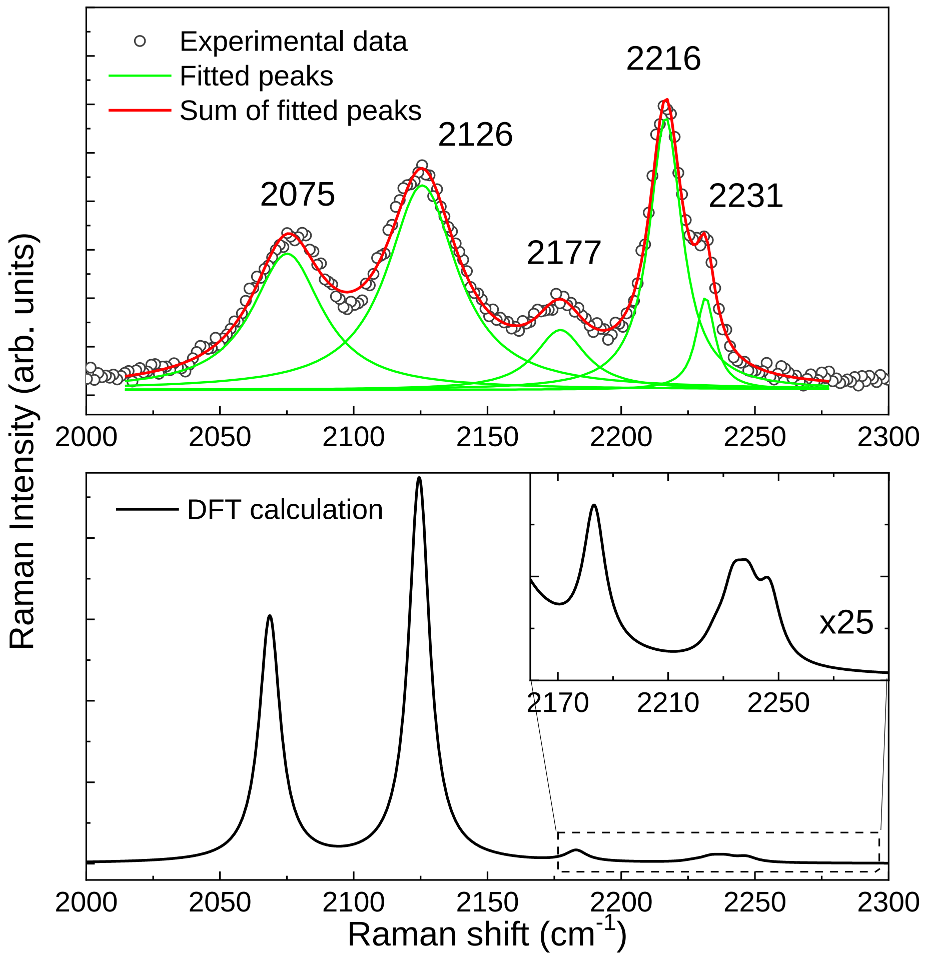

Figure S4: (Top) sp-carbon region detail of the experimental FT-Raman spectrum of C6/TCBD (1064 nm, in film) and fit of the contribution. (Bottom) DFT calculation of the same region.

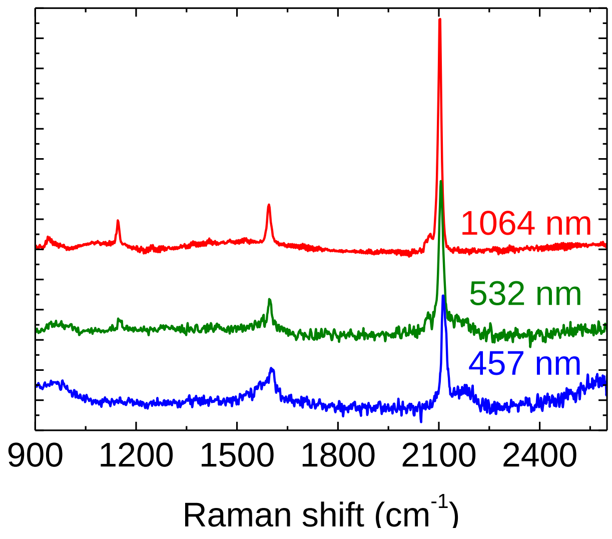

Figure S5: Raman spectra at different wavelengths of C8 deposited in film.

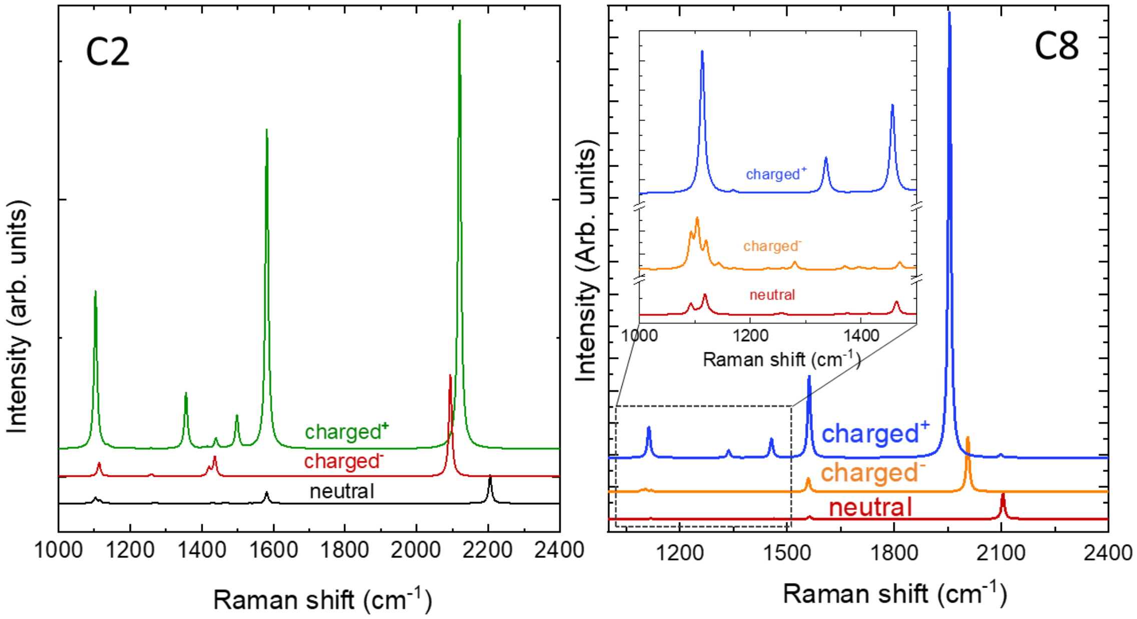

Figure S6: Comparison of DFT computed Raman spectra of neutral, positively and negatively charged C2 (left) and C8 (right) molecule. Details of the region at low frequency (1000-1500 cm^{-1}) is reported in the inset.

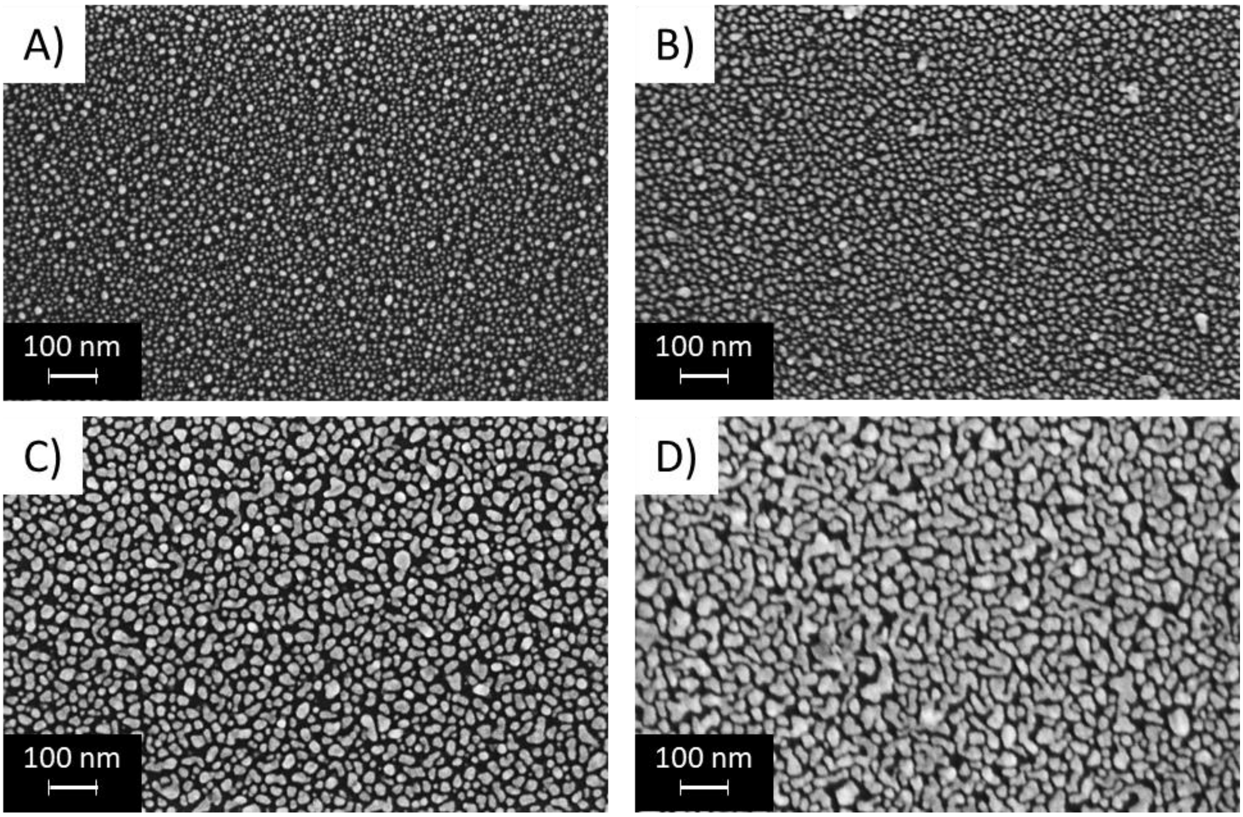

Figure S7: Morphological SEM images of solid SERS-active substrates obtained by vacuum deposition of silver nanoislands.

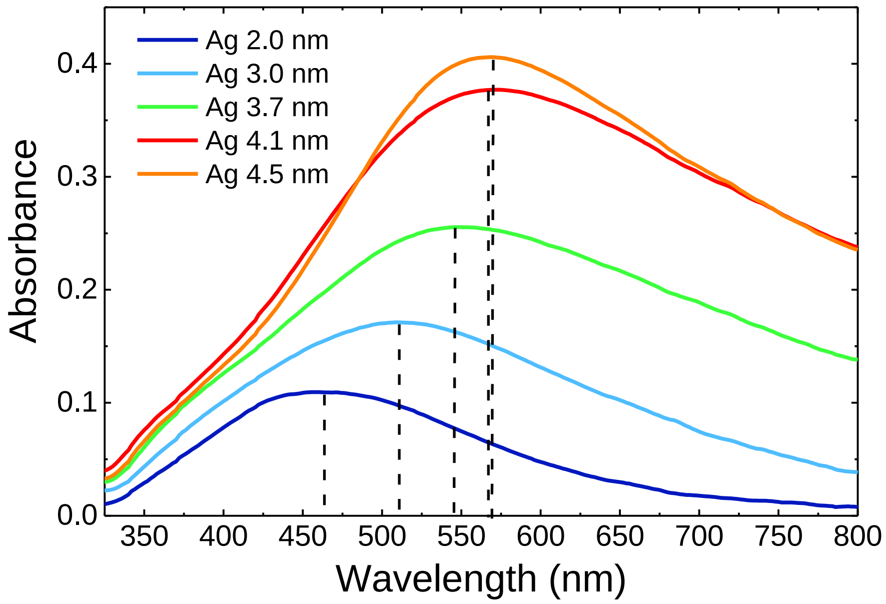

Figure S8: UV-vis absorption spectra of Ag SERS active substrates on glass.

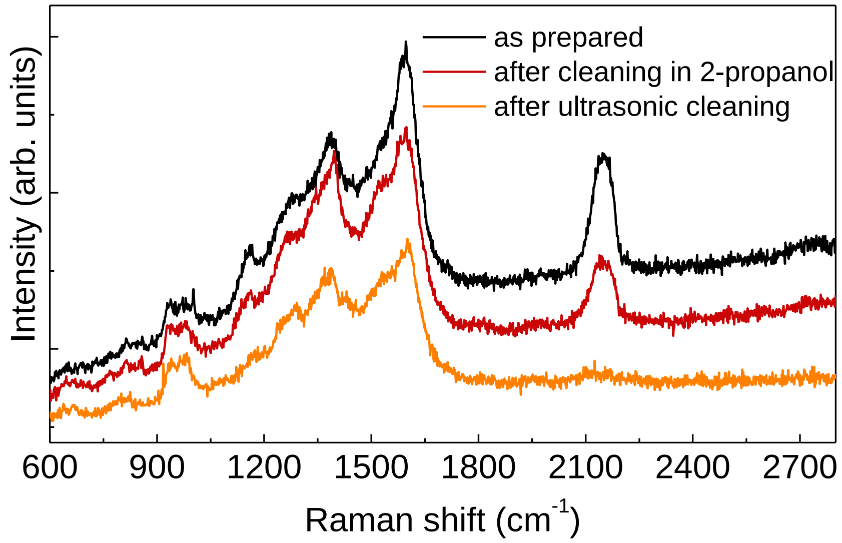

Figure S9: Raman spectra of bare SERS-active substrates (3.0 nm Ag equivalent thickness) as prepared and after different cleaning procedures.

Table S1: DFT computed values of E_{ion} in eV computed for the investigated systems in the two configurations Ag^+/CAW^-, Ag^-/CAW^+; $\Delta E_{ion} = E_{ion}(Ag^+/CAW^-) - E_{ion}(Ag^-/CAW^+)$ is also reported. The values of the BLA (bond length alternation) are reported for neutral and charged systems.

Molecule	Ag^+/CAW^- [eV]	Ag^-/CAW^+ [eV]	ΔE_{ion} [eV]	BLA-neutral [Å]	BLA-charged [Å]
C2	4.329	4.028	+0.301	-	-
C4	3.898	4.031	–0.133	0.1424	0.0973
C6	3.522	4.040	–0.518	0.1323	0.0888
C8	3.050	4.051	–1.001	0.1257	0.0842
C6/TCBD	1.840	5.559	–3.719	0.1167	0.1220
